# Compound Probiotics Alleviate Gut Microbiota Dysbiosis Induced by Heat Stress in Broilers

**DOI:** 10.3390/ani16050823

**Published:** 2026-03-06

**Authors:** Fenghua Li, Panping Sun, Muchun Duan, Xuan Liu, Lihuan Zhang

**Affiliations:** College of Life Sciences, Shanxi Agricultural University, Jinzhong 030801, China; lfenghua1229@163.com (F.L.); spanping@163.com (P.S.); 18834237058@163.com (M.D.); lxuan0919@163.com (X.L.)

**Keywords:** probiotics, heat stress, broiler, cecal microbiota, 16S rDNA amplicon sequencing

## Abstract

Heat stress represents a key environmental challenge in poultry production, markedly impairing broiler health and productivity. This study investigated the association between compound probiotic supplementation and the gut microbial community structure in heat-challenged broilers, analyzing the cecal contents from both groups using 16S rDNA amplicon sequencing. Compound probiotic supplementation was associated with changes in alpha diversity and richness of the cecal microbiota, with lower Shannon, Chao1, and ACE indices (*p* < 0.05). At the phylum level, compound probiotic supplementation significantly increased the relative abundance of *Bacteroidota* (*p* < 0.001) while decreasing that of *Proteobacteria* (*p* < 0.0001) in the cecum of broilers, whereas the relative abundances of *Firmicutes* and *Verrucomicrobiota* showed increasing trends. At the genus level, the relative abundance of *Bacteroides* (*p* < 0.0001) was significantly increased in the HP group, whereas *Lactobacillus* and *Fusobacterium* exhibited decreasing trends compared with the HS group. LEfSe analysis suggested *Verrucomicrobia* as a potentially enriched taxon in the HP group. Furthermore, KEGG level 3 functional prediction suggested enrichment of predicted pathways related to starch and sucrose metabolism, as well as amino acid and nucleotide metabolism in the HP group. These findings suggest that compound probiotics are associated with changes in gut microbial composition and predicted functions in heat-stressed broilers, providing preliminary, exploratory insights into their potential associations under heat stress.

## Simple Summary

The detrimental impact of high temperatures represents a major risk factor for both the health status and meat production efficiency in commercial broilers. This study investigated the potential associations between feeding broilers a mix of beneficial bacteria (compound probiotics) and changes in the gut microbial community under heat stress. Compound probiotic supplementation significantly increased the relative abundance of *Bacteroidota* while reducing that of *Proteobacteria* in the gut microbiota of broilers. These changes were associated with predicted enrichment of pathways related to carbohydrate and amino acid metabolism, suggesting potential metabolic alterations under heat stress. Overall, these findings provide preliminary, exploratory insights into potential associations between compound probiotics and gut microbial composition in heat-stressed broilers, offering guidance for future research in poultry production.

## 1. Introduction

As one of the most widely raised livestock species globally, the broiler is a major source of dietary protein [1,2]. The consumption of poultry meat has steadily increased globally over the past few decades [3]. Poultry farming is critical to meeting the worldwide food demand due to the increasing population [4]. With the ongoing rise in global average temperatures, heat stress is increasingly recognized as a key factor affecting poultry productivity [5,6]. Broilers exhibit pronounced susceptibility to heat stress because of their restricted heat dissipation ability, elevated metabolic rates, high body temperatures, narrow thermal tolerance range, absence of sweat glands, and feather covering. Heat stress in poultry can induce hyperthermia, immune system suppression, impairment of intestinal epithelial structure and function, and dysbiosis of the intestinal microbiota. Therefore, heat stress can adversely affect broiler production, leading to diminished growth performance, increased mortality, and considerable economic losses [7,8]. Evidence indicates that ecological dysregulation due to heat stress may lead to metabolic inefficiency and heightened intestinal permeability [9].

The digestive system, as one of the primary immunological and neuroendocrine organs in broilers, is highly susceptible to heat stress. It also serves a crucial function in nutrient absorption, both of which are essential for broiler production [10]. Gut microbial diversity and community structure are shaped by multiple factors, such as the intestinal region, host age, genetic makeup, feeding practices, and dietary composition [11]. The microflora in the cecum, the most diverse region of the gastrointestinal tract microbiota in broilers, is crucial for promoting growth and regulating gut function [12]. The cecum is the main location for short-chain fatty acids (SCFAs) production. SCFAs are known to benefit broiler health by supporting growth performance, increasing nutrient utilization efficiency, and influencing fat deposition in both body and muscle tissues [13]. Heat stress–related shifts in intestinal microbiota and SCFA generation are thought to contribute to metabolic disorders that underlie impaired growth in broilers [14]. The complex microbiota residing in the cecum can ferment indigestible fibers to produce several fermentation products, including SCFAs, which are crucial for sustaining intestinal barrier integrity [15]. The gastrointestinal system is highly vulnerable to multiple types of stress, including heat stress. Heat exposure is an ongoing concern in poultry production, as it disrupts physiological homeostasis and adversely affects the health, growth, immune function, and intestinal structure of chickens [16]. These stressors can damage the intestinal barrier, alter epithelial cell morphology, increase the vulnerability of commensal bacteria to infection, and potentially lead to intestinal diseases [17]. These adverse effects are thought to arise partly from disturbances in the gut microbiota balance, which may increase intestinal permeability and trigger immune and metabolic dysfunctions [18,19].

While heat stress compromises intestinal integrity and increases disease susceptibility in broilers, the concurrent ban on antibiotic growth promoters further limits effective interventions, highlighting the need for alternative strategies [20]. Such disturbances in intestinal integrity and microbial composition arising from heat stress emphasize the importance of nutritional interventions that can restore intestinal balance and sustain growth without relying on antibiotic growth promoters. Among the promising alternatives, probiotics have attracted attention for their ability to improve intestinal well-being and enhance productive output [21,22,23]. Probiotics are live microorganisms that help maintain gastrointestinal microbial balance, suppress pathogenic bacteria, enhance digestion and nutrient absorption, and boost the immune response in broilers [24]. Probiotics improve immune function, gut barrier integrity, nutrient absorption, and intestinal microbial richness [25]. Probiotics have demonstrated efficacy in counteracting the deleterious consequences of thermal burden and are increasingly employed as alternative therapies to promote animal health [26,27]. Probiotics, such as *Lactobacillus acidophilus*, *Bifidobacterium* spp., *Propionibacterium* spp., *Aspergillus* spp., and *Bacillus* spp., are commonly employed in chicken husbandry [28]. *Lactobacillus* species and their metabolites inhibit intestinal pathogens and promote beneficial bacteria such as *Bifidobacterium bifidum* [29].

Multistrain probiotic complexes may provide synergistic effects that surpass those of single strains, offering broader protection under thermal stress conditions [30]. Beneficial gut microbes, including *Bifidobacterium*, *Lactobacillus casei*, and *Lactobacillus acidophilus*, contribute significantly to broiler health [31]. Previous research indicates that *Bifidobacterium bifidum*, *Lactobacillus casei*, and *Lactobacillus acidophilus* may influence lipid biosynthesis and production performance in broilers under thermal stress [32]. Compared with single strains, compound probiotics generally have stronger effects, modulating the intestinal microbiota more effectively under heat stress conditions [33,34,35]. Research indicates that modifying the intestinal microbiota of broilers with a probiotic complex containing *Bacillus subtilis*, *Lactobacillus casei*, and *Pseudomonas syringae* can significantly enhance intestinal health and improve broiler performance [36]. Moreover, *Bacillus subtilis* is able to reduce heat-provoked behavioral and inflammatory disturbances by influencing microbiota-associated immune regulation [37]. Recent research indicates that dietary supplementation with a probiotic complex under heat stress enhances both growth performance and antioxidant capacity in broilers [38].

To meet consumer demand and ensure safety, a compound probiotic was formulated by combining *Lactobacillus casei*, *Lactobacillus acidophilus*, and *Bifidobacterium* in a 1:1:2 ratio. This research focused on assessing the impact of supplementing this composite probiotic on the compositional architecture and predicted metabolic activity of the cecal microbiota in heat-exposed broilers. We hypothesize that compound probiotic supplementation can modulate gut microbial composition and predict functional potential under heat stress, thereby alleviating heat-induced microbial dysbiosis.

## 2. Materials and Methods

### 2.1. Examination Resources

The probiotics utilized in the present research were obtained from Shaanxi Ruimao Biotechnology Co., Ltd. (Xi’an, China) and comprised freeze-dried bacterial powder (*Lactobacillus casei*, *Lactobacillus acidophilus*, and *Bifidobacterium*) with a viable bacterial concentration of 1.0 × 10^10^ CFU/g. The probiotics were formulated in a 1:1:2 ratio to generate a composite probiotic. The composition and ratio of the compound probiotics (1:1:2) were selected based on our previously published optimization study. The strains were selected based on their stability during feed processing and under environmental conditions.

### 2.2. Animal Nutrition and Experimental Methodology

Three hundred one-day-old Arbor Acres broilers were obtained from Xiangfeng Poultry Industry (Jinzhong, China). At 28 days of age, 200 broilers with similar body weights were randomly allocated to two treatment groups. The heat stress with probiotic supplementation (HP) group was administered a basal diet supplemented with 10 g/kg of a compound probiotic, whereas the heat stress control (HS) group received only the standard diet without probiotic supplementation. For each treatment, five replicates were established, each comprising 20 broilers. The inclusion level of 10 g/kg was selected based on our previous research, which evaluated 1, 5, and 10 g/kg supplementation and found that 10 g/kg was associated with more favorable outcomes in terms of physiological development and intestinal-related parameters in broilers. Based on the recorded feed intake during the experimental period, the estimated daily probiotic intake was approximately 1.68 × 10^10^ CFU/bird in the HP group.

Broilers were maintained in stacked three-level cages, where feed and water were available without limitation, and the housing units underwent regular disinfection. Broilers were reared according to a conventional two-phase feeding program, comprising a starter period from days 1 to 21 and a grower period from days 22 to 42. The dietary treatments were introduced from day 28 to day 42 during the grower phase. The trial lasted fourteen days. We refreshed the drinking water every eight hours without disturbing the broilers. Broilers had ad libitum access to feed, which was replenished twice daily at 08:00 and 20:00 to ensure continuous availability. To guarantee continuous feeding and drinking, the broilers were kept under a 23 h light and 1 h dark cycle. The temperature was kept at 34 ± 1 °C for the initial three days and subsequently reduced to 32 ± 1 °C between days 4 and 7 post-hatching. Thereafter, the temperature was reduced by 1 °C per day until it stabilized at 21 ± 1 °C. We documented the broilers’ health conditions daily. Beginning on day 28, broilers across all groups were continuously exposed to heat throughout the duration of the experiment. The chicken housing temperature was maintained at 33 ± 1 °C daily between 9:00 and 17:00, with heat applied for 8 h. Throughout the remainder of the experimental period, the ambient temperature was maintained at 21 ± 1 °C. Broiler rearing and management complied with the national standard GB/T 19664-2005: Technical Specification for the Production of Commercial Broiler Chickens. We provided the groups with a basic diet that included maize and soybean meals. The corn-soybean meal formulation was developed in accordance with the guidelines set forth by the National Research Council. Table 1 provides the nutritional composition of the basal diet.

### 2.3. Sample Collection and Analysis

Broilers were fasted for 12 h with free access to water prior to sampling on days 21 and 42. At 42 days, the broilers were euthanized and dissected, and samples of cecal contents were carefully removed. Specifically, four independent biological replicates per group were collected, with each sample obtained from one individual broiler selected from different experimental replicates. The samples were immediately frozen in liquid nitrogen and then stored at −80 °C to preserve their integrity. The cecal content samples were dispatched to Beijing Novozymes Biotechnology Co., Ltd. (Beijing, China) for bacterial 16S rDNA sequencing to obtain comprehensive data on cecal microbiota and facilitate in-depth biological analysis.

### 2.4. Amplification and Sequencing of 16S rDNA

To analyze the gut microbiota, four cecal content samples per group were randomly collected from 42-day-old broilers in both the heat stress control and HP groups for 16S rDNA amplification and sequencing. Barcode-specific sequences were incorporated into the 341F (CCTAYGGRBGACAG) and 806R (GGACTACNNGGTATCTAAT) primers, which are designed to amplify the V3–V4 region of the 16S rDNA gene to enable sample multiplexing. PCR amplification was performed, followed by library construction and quantification of the libraries. Sequencing was performed using the NovaSeq 6000 platform. Post-sequencing, sequences with fewer than five copies were filtered out using the DADA2 plugin in QIIME2 to obtain the final amplicon sequencing variants (ASVs). The abundance of each ASV was quantified by taxonomic classification using the sklearn classifier module in QIIME2 (version 2020.6). ASVs were taxonomically assigned by comparison against the reference database.

### 2.5. Bioinformatics Assessment

QIIME2 software was employed to calculate the Shannon, Simpson, Chao1, and ACE indices, and rarefaction curves were generated to assess the sequencing depth. Venn diagrams, PCA plots, and bar charts were used to show the composition and distribution of microorganisms by displaying the relative amounts of species within the top ten phyla and genera. The Linear Discriminant Analysis Effect Size (LEfSe) analysis tool was utilized with an LDA score threshold of 4.0 to identify microbial taxa exhibiting significant differences between groups. Furthermore, to understand microbial functions, we employed PICRUSt functional prediction analysis to acquire annotated data regarding microorganisms from the Kyoto Encyclopedia of Genes and Genomes (KEGG) database. Downstream statistical analyses were performed using the R program (version 3.5.3).

### 2.6. Statistical Analysis of Data

All statistical analyses were carried out with SPSS (version 26.0). Statistical differences among groups were assessed by one-way analysis of variance (ANOVA) with Duncan’s multiple range test, and significance was set at *p* < 0.05. Differences between the two groups were analyzed using an independent-samples *t*-test.

## 3. Results

### 3.1. Analysis of the Differences in the Intestinal Microbiota Between Groups

**Figure 1:**
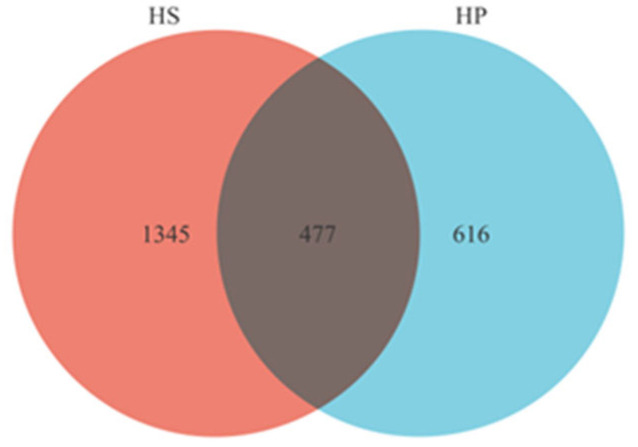
Venn diagram of cecum intestinal microflora counts (*n* = 4). Note: HS: broilers were exposed to 33 ± 1 °C from 09:00 to 17:00 at 28 days, maintained at 21 ± 1 °C for the remainder of the period, receiving a basal diet without probiotic supplementation. HP: broilers received a basal diet supplemented with compound probiotics (10 g/kg) under the same heat stress regimen as the HS group.

Figure 1 depicts the variations in gut microbial composition between the HS and HP groups. The HS and HP groups shared 477 ASVs, with the HS group exhibiting 1345 unique ASVs and the HP group displaying 616 unique ASVs. These results showed that heat stress was associated with a higher number of detectable microbial taxa in the cecum of broilers, whereas probiotic supplementation was associated with a lower number of unique ASVs, reflecting differences in cecal microbial community structure between the groups.

**Figure 2:**
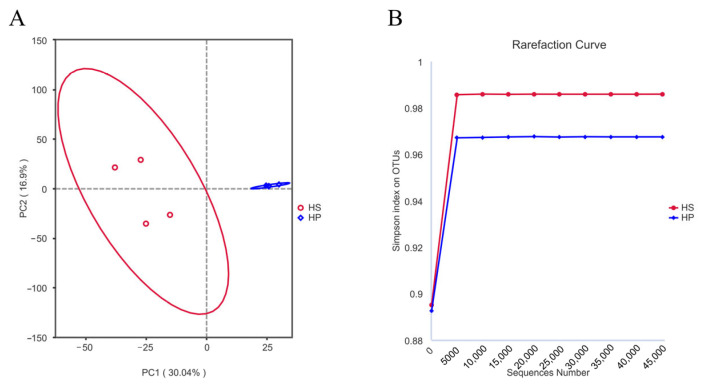
PCA and rarefaction curves (*n* = 4). Note: (**A**,**B**) represent the PCA and dilution curves of the HP group compared with the HS group. The ellipse represents the 95% confidence interval for each group.

Figure 2 illustrates the PCA plot between groups alongside the rarefaction curve represented by Simpson’s index. Figure 2A clearly shows a difference between the HS and HP groups, indicating reliable sequencing data and valid grouping. Figure 2B illustrates that the rarefaction curve, as determined by Simpson’s index, exhibits a tendency to plateau with increasing sequencing depth. This plateau occurs when the sample size is adequate, and the sequencing depth approaches saturation, indicating that further sequencing is unlikely to yield new species.

### 3.2. Alpha Diversity of the Gut Microbiota

**Figure 3:**
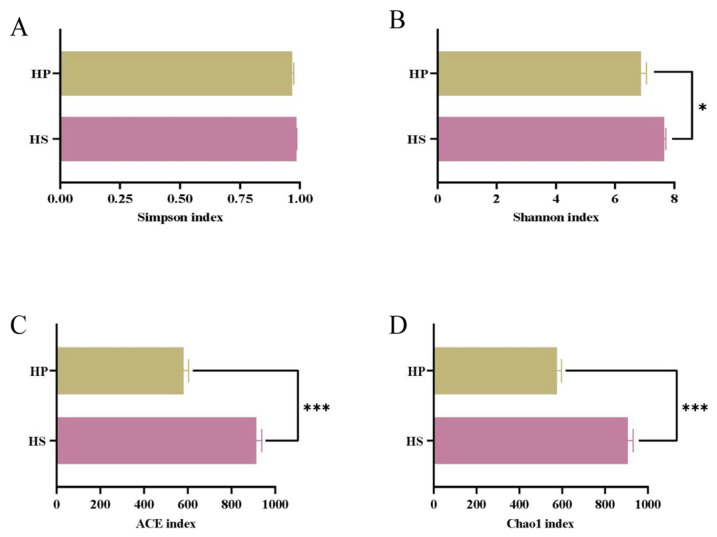
Effects on the Alpha diversity of the intestinal microbiota (*n* = 4). Note: (**A**) Simpson index; (**B**) Shannon index; (**C**) ACE index; (**D**) Chao1 index. The data are presented as M + SEM, * *p* < 0.05 and *** *p* < 0.001.

Microbial diversity was quantified via the Shannon and Simpson indices, whereas richness was evaluated based on the Chao1 and ACE estimators. In this research, the HS group served as a control. Figure 3 illustrates that compound probiotic supplementation reduced cecal microbial diversity and richness in the HP group, as indicated by significantly lower Shannon (Figure 3B), Chao1 (Figure 3D), and ACE (Figure 3C) indices (*p* < 0.05); however, the Simpson (Figure 3A) index did not differ significantly (*p* > 0.05).

### 3.3. Compositional Analysis of the Intestinal Microbiota

**Figure 4:**
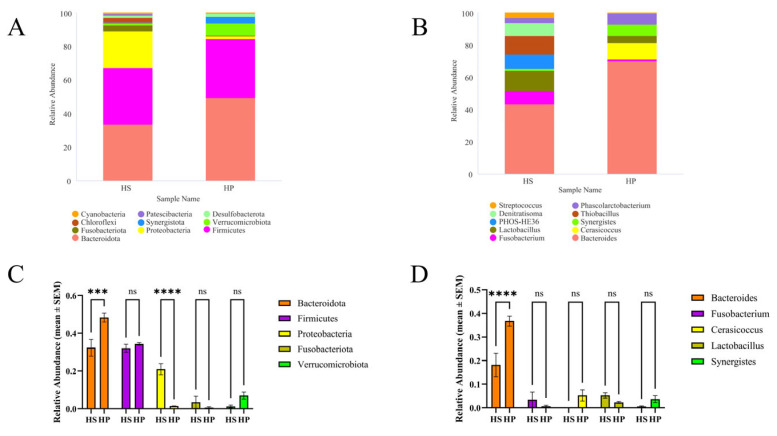
Impact on the composition of the intestine microbiota community structure (*n* = 4). Note: Panel (**A**) illustrates the ten most abundant taxa at the phylum level, whereas panel (**B**) depicts the ten most abundant taxa at the genus level based on relative abundance. Panels (**C**,**D**) show the relative abundances of the five most abundant phyla and genera, respectively, with statistical comparisons between groups. Data are presented as mean ± SEM, *** *p* < 0.001 and **** *p* < 0.0001; ns indicates no significant difference.

Figure 4A illustrates changes in the relative proportions of the top ten bacterial phyla within the cecal microbiota. In the cecum of broilers from the HS and HP groups, *Bacteroidota* and *Firmicutes* exhibited the highest proportional abundance, establishing them as the predominant phyla at 42 days of age. Compared with the HS group, broilers in the HP group showed a significant increase in the relative abundance of *Bacteroidota* (*p* < 0.001) and a significant decrease in the relative abundance of *Proteobacteria* (*p* < 0.0001), whereas the relative abundances of *Firmicutes* and *Verrucomicrobiota* showed an increasing trend, and *Fusobacteriota* showed a decreasing trend, although these changes were not statistically significant (Figure 4C).

Figure 4B shows that at the genus taxonomic level, *Bacteroides* was the dominant genus in both groups. Compared with the HS group broilers, broilers in the HP group exhibited a significant increase in the relative abundance of *Bacteroides* (*p* < 0.0001), while the relative abundances of *Lactobacillus* and *Fusobacterium* showed a decreasing trend, without reaching statistical significance (Figure 4D).

### 3.4. Comparative Analysis of Various Microorganisms Among Groups

**Figure 5:**
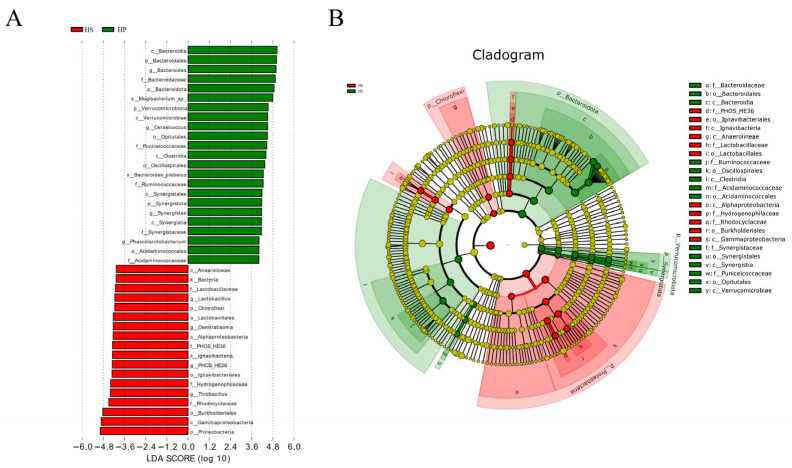
Effects on differential microbiota (*n* = 4). Note: (**A**) Species with a linear discriminant analysis (LDA) score greater than 4 are displayed in histograms where the bar lengths represent their respective effect sizes. (**B**) The generated cladogram displays concentric circles emanating from the center, illustrating hierarchical taxonomic levels spanning from phylum to genus (or species). The node size reflects the representation of the corresponding taxon at the taxonomic level. Species with no significant differences are uniformly colored in yellow, and differential species are colored according to the group.

Microorganisms showing differential abundance between groups were explored using LEfSe analysis, with an LDA threshold of 4, as presented in Figure 5. LEfSe analysis identified multiple taxa showing differential enrichment across different taxonomic levels, with 23 taxa relatively enriched in the HP group and 18 taxa in the HS group. The levels of *p_Bacteroidota*, *g_Bacteroides*, *g_Prevotella*, *f_Veillonellaceae*, and *g_Veillonella* in the HP group were appreciably greater than those in the HS group, whereas *p_Proteobacteria*, *g_Desulfovibrio*, *g_Enterococcus*, *g_Akkermansia*, and *c_Actinobacteria* were lower.

Figure 5A illustrates an association of *p_Proteobacteria* and *p_Fusobacteriota* with the HS group, whereas *c_Bacteroidia*, *p_Verrucomicrobiota*, and *g_Cerasicoccus* were more closely associated with the HP group. As shown in Figure 5B, cluster analysis was conducted to determine the distinctive taxonomic characteristics of each group from the phylum to the genus level. The results indicated that the microorganisms predominantly associated with the HS and HP groups were classified primarily within *p_Proteobacteria* and *p_Bacteroidota*, respectively. The phylogenetic analysis further suggested that the microorganisms in the HP group were related mainly to *p_Bacteroidota*, *p_Verrucomicrobiae*, and *c_Synergistia*, whereas those in the HS group were related mostly to *p_Proteobacteria*, *o_Lactobacillales*, and *p_Chloroflexi*.

Evolutionary analyses showed that *c_Bacteroidia* and *o_Bacteroidales* and *f_Bacteroidaceae* belong to *p_Bacteroidota*, *c_Verrucomicrobiae* and *o_Opitutales* and *f_Puniceicoccaceae* belong to *p_Verrucomicrobiota*, *o_Synergistia* and *f_Synergistaceae* belong to *c_Synergistia*, whereas *c_Anaerolineae* belongs to *p_Chloroflexi*, *f_Lactobacillaceae* belongs to *o_Lactobacillales*, *c_Gammaproteobacteria* and *c_Alphaproteobacteria* and *o_Burkholderiales* and *f_Rhodocyclaceae* belong to *p_Proteobacteria*.

**Figure 6:**
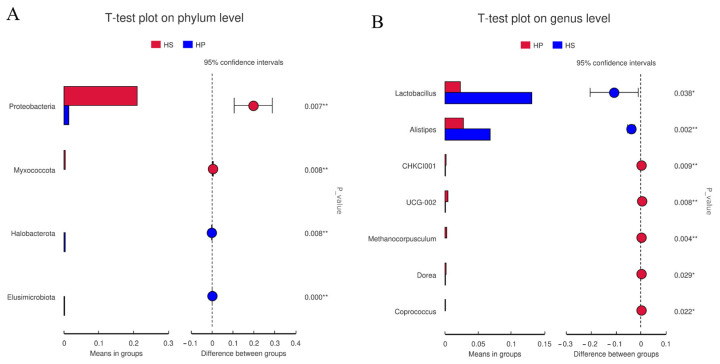
Microbiological analysis of differences between groups (*n* = 4). Note: (**A**) At the phylum level, the left figure shows the between-group difference in species abundance, the right figure shows the between-group difference confidence level, the leftmost endpoint of each circle in the graph represents the lower 95% confidence interval of the mean difference, the rightmost endpoint of the circle represents the upper limit of the 95% confidence interval of the mean difference, the center of the circle represents the difference in the mean, and the color of the circle represents the significance test *p*-value of the difference between groups of the corresponding different species. (**B**) At the genus level. The data are presented as mean ± SEM, * *p* < 0.05 and ** *p* < 0.01.

As illustrated in Figure 6, at the phylum level, the relative abundance of *p_Proteobacteria* was significantly reduced in the HP group compared with the HS group. At the genus level, the relative abundances of *g_Lactobacillus* and *g_Alistipes* were markedly reduced, whereas that of *g_UCG-002* was significantly elevated following probiotic supplementation.

### 3.5. Predictive Analysis of Gut Microbial Functional Potential

**Figure 7:**
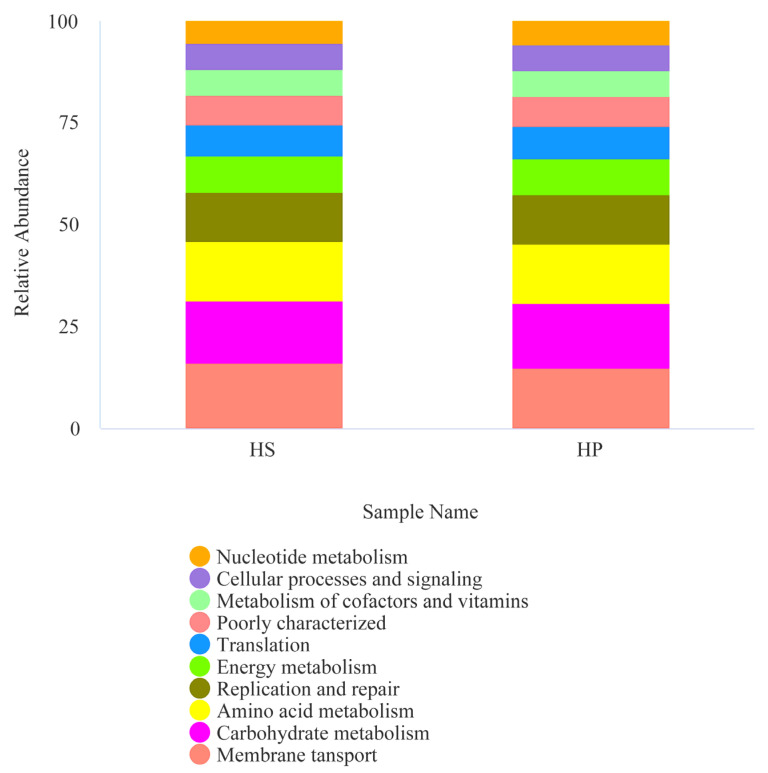
KEGG level 2 horizontal function prediction (*n* = 4). Note: The PICRUSt function predicts the top 10 stacking maps at level 2.

To investigate the predicted functional potential of the gut microbiota, KEGG level 2 pathway analysis indicated that the broiler cecal microbiota in the HS and HP groups were predominantly enriched in the top 10 functional categories, including pathways related to nucleotide and energy metabolism, cellular processes and signaling, vitamin and cofactor biosynthesis, replication and repair, and amino acid and carbohydrate utilization. Moreover, distinct differences in predicted microbial functional profiles were observed between the groups (Figure 7).

**Figure 8:**
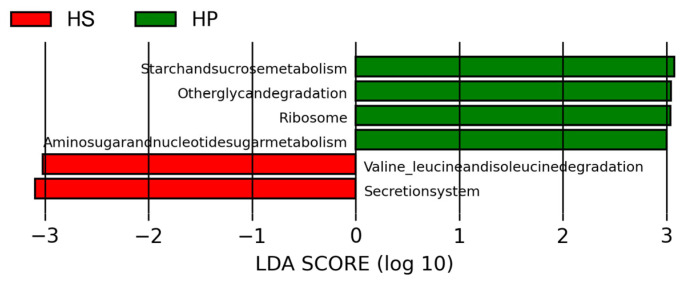
KEGG level 3 horizontal function prediction (*n* = 4). Note: Metabolic pathways with LDA values greater than 3 are illustrated in the LDA value distribution histograms, where the histogram lengths denote the size of the effects of the distinct metabolic pathways.

To further elucidate the predicted microbial functional differences among the groups, LEfSe analysis was conducted with an LDA threshold of 3.0 (Figure 8). Compared with the HS group, the cecal microbiota of the HP group showed higher predicted abundance of several KEGG level 3 pathways, including ribosome, starch and sucrose metabolism, other glycan degradation, and amino sugar and nucleotide sugar metabolism. In contrast, the HS group was characterized by a higher predicted abundance of pathways related to valine, leucine, and isoleucine degradation and the secretion system pathway.

## 4. Discussion

Multiple studies have indicated that gut microbial balance plays an important role in intestinal function and overall health in broilers [39,40,41,42]. Balanced gut microbial communities are vital for regulating intestinal function and microbial stability. Goel et al. [43] indicated that the provision in the feed of probiotics reduced the number of OTUs and lowered the Chao1, Shannon, and Simpson indices in heat-stressed chickens. The findings of this investigation align with those of previous studies, showing that the administration of a compound probiotic in the diet was associated with a reduction in ASVs and the Chao1, ACE, and Shannon indices in heat-stressed broilers. Importantly, a reduction in alpha diversity does not necessarily indicate improved gut health but may instead reflect microbial restructuring under the pressure of compound probiotic supplementation. Moreover, extreme heat stress can substantially disrupt intestinal microbial equilibrium in broilers, leading to ecological disturbances [44]. Conversely, probiotic supplementation partially mitigated changes in microbial diversity and abundance in the cecum of broilers under heat stress [45,46,47,48]. These results indicate that compound probiotic supplementation was associated with notable alterations in cecal microbial diversity in heat-stressed broilers.

Furthermore, our findings indicate that the cecal microbiota composition in broilers receiving compound probiotics underwent considerable alterations under heat stress. Probiotics have been reported to enhance the proliferation of beneficial intestinal microbes while inhibiting the expansion of potentially unfavorable bacterial strains [49,50,51]. In this investigation, the microbial profiles differed significantly between the HS and HP groups at both taxonomic levels. At the phylum level, compound probiotic supplementation significantly increased the relative abundance of *Bacteroidota* and decreased that of *Proteobacteria* in the cecal microbiota of broilers. In contrast, the relative abundances of *Firmicutes* and *Verrucomicrobiota* showed increasing trends. At the genus level, the relative abundance of *Bacteroides* was significantly increased in the HP group compared with the HS group. In addition, the relative abundances of *Synergistes* and *Cerasicoccus* showed increasing trends, whereas *Lactobacillus* and *Fusobacterium* exhibited decreasing trends. The decrease in *Lactobacillus* likely reflects the expansion of other bacterial taxa, such as *Bacteroides* and *Verrucomicrobia*, promoted by the compound probiotic, resulting in a proportional reduction in *Lactobacillus* in this gut region. LEfSe analysis indicated that *Verrucomicrobia* tended to be more abundant in the HP group. Based on KEGG level 2 analysis, the dominant microbial functions in the broiler cecum were predicted to be linked to nucleotide and amino acid metabolism, cellular signaling, energy pathways, genetic information processing, and the biosynthesis of cofactors, vitamins, and carbohydrates. Furthermore, KEGG level 3 functional prediction showed that starch and sucrose metabolism, together with the metabolic processes of amino acids and nucleotides, may be more pronounced in the HP group. *Verrucomicrobia* have been suggested to play roles in organic carbon degradation and antimicrobial activity, and may be involved in microbial nutrient metabolism under conditions of limited intestinal nutrients [52,53]. Previous studies have reported that higher levels of *Verrucomicrobia* may be associated with beneficial changes in the gut microbial community in laying hens [54]. *Phascolarctobacterium* is a consistent member of the broiler cecal microbiota and may contribute to gut health and immune development [55]. Therefore, the increased relative abundances of *Verrucomicrobia* and *Phascolarctobacterium* observed in this study suggest that compound probiotics are associated with alterations in the gut microbial ecosystem. Collectively, these microbial changes suggest that compound probiotics modulate the gut microbial ecosystem in heat-stressed broilers.

Compound probiotics stimulate the expansion of health-promoting intestinal microbes and suppress the proliferation of potentially pathogenic taxa. Zhu et al. reported that nutritional supplementation with heat-killed *Bacillus subtilis* and *Lactobacillus acidophilus* significantly enriched the microbial community from the cecum of broilers at 42 days of age, with notable enrichment in metabolic pathways related to starch and sucrose utilization, amino acid turnover, and nucleotide metabolism [56]. Previous studies have reported that these pathways are enriched in the cecal microbiota of broilers, suggesting potential associations with microbial metabolic functions [57,58,59]. In this context, the compound probiotics used in the present study may be associated with changes in microbial metabolic pathways related to starch, sucrose, and amino acid metabolism. Taken together, these increases in beneficial bacteria and changes in nutrient-related metabolic pathways support our hypothesis that compound probiotic supplementation may partially mitigate heat stress–induced alterations in the intestinal microbiome of broilers. Although the predicted KEGG-based functional analysis provides preliminary insights into potential microbial functional shifts, a limitation of this study is that microbial functions were inferred using PICRUSt rather than directly measured, and host physiological or metabolic outcomes, including growth performance and SCFA levels, were not assessed. Consequently, the KEGG-based functional predictions should be considered tentative. Future studies incorporating direct metabolite measurements and host performance data are warranted to validate these observations.

## 5. Conclusions

In summary, compound probiotics were associated with changes in the intestinal microbiota of heat-stressed broilers, including alterations in microbial diversity and the relative abundance of potentially beneficial bacteria such as *Bacteroides*. They were also linked to predicted enrichment of microbial metabolic functions related to carbohydrate utilization, amino acid turnover, and nucleotide biosynthesis, reflecting shifts in microbial metabolic potential. Overall, these findings provide preliminary, exploratory insights into the gut microbial ecosystem under heat stress and highlight potential associations between compound probiotics and intestinal microbial communities. This work may inform future research aimed at understanding and mitigating the effects of thermal stress in poultry systems.

## Figures and Tables

**Table 1:** Basic feed composition and nutritional level.

Items	11 to 21 Days of Age	22 to 42 Days of Age
Ingredients		
Corn	56.49	61.42
Soybean oil	2.22	3.00
Soybean meal	30.24	25.30
Cottonseed meal	5.00	5.00
Fish meal	2.43	1.98
CaHPO_4_	1.60	1.39
Limestone	1.16	1.10
Met	0.15	0.05
NaCl	0.30	0.35
Choline	0.19	0.19
Premix ^a^	0.22	0.22
Total	100.00	100.00
Nutrient levels ^b^		
ME (MJ/kg)	12.12	12.54
Crude protein	21.00	19.00
Lysine	1.12	0.98
Methionine + Cystine	0.84	0.68
Calcium	1.00	0.90
Available phosphorus	0.30	0.30

Note: ^a^ Premix provided the following per kg of diets: Fe 50 mg, Mn 65 mg, Zn 45 mg, Cu 8 mg, I 0.35 mg, Se 0.15 mg, VA 12,000 IU, VD3 3000 IU, VE 30 IU, VK3 1 mg, VB1 2 mg, VB2 6 mg, Pantothenic acid 9 mg, Pyridoxine 5 mg, Niacin 30 mg, VB12 0.01 mg, Biotin 0.10 mg, Folic acid 0.30 mg. ^b^ Nutrition levels were calculated value.

## Data Availability

The data presented in this study are available on request from the corresponding author upon reasonable request.

## Abbreviations

The following abbreviations are used in this manuscript:

ANOVA	Analysis of Variance
ASVs	Amplicon Sequencing Variants
LDA	Linear Discriminant Analysis
KEGG	Kyoto Encyclopedia of Genes and Genomes
LEfSe	Linear Discriminant Analysis Effect Size
OTUs	Operational Taxonomic Units
SCFAs	Short-Chain Fatty Acids
